# The protective effects of rutin on the liver, kidneys, and heart by counteracting organ toxicity caused by synthetic and natural compounds

**DOI:** 10.1002/fsn3.3041

**Published:** 2022-09-15

**Authors:** Sohrab Rahmani, Karim Naraki, Ali Roohbakhsh, A. Wallace Hayes, Gholamreza Karimi

**Affiliations:** ^1^ Student Research Committee Mashhad University of Medical Sciences Mashhad Iran; ^2^ Department of Pharmacodynamics and Toxicology, School of Pharmacy Mashhad University of Medical Sciences Mashhad Iran; ^3^ Pharmaceutical Research Center, Institute of Pharmaceutical Technology Mashhad University of Medical Sciences Mashhad Iran; ^4^ Center for Environmental Occupational Risk Analysis and Management, College of Public Health University of South Florida Tampa Florida USA; ^5^ Institute for Integrative Toxicology Michigan State University East Lansing Michigan USA

**Keywords:** apoptosis, flavonoid, oxidative stress, rutin

## Abstract

Rutin is a flavonoid present in many plant species. Because of its antioxidant, anti‐inflammatory, and antiapoptotic properties, rutin is of interest for its potential protective effects against toxic agents. The hepatoprotective, renoprotective, and cardioprotective effects of rutin are reviewed. The antioxidant effects of rutin are elicited by enhancing antioxidant enzymes such as GST, GGT, CAT, GPx, SOD, and GR, activating the Nrf2/HO‐1 pathway, elevating GSH content, and the reduction in MDA. The anti‐inflammatory effects of rutin are mediated by the inhibition of IL‐1β, IL‐6, TGF‐β1, COX‐2, iNOS, TLR4, and XO. Rutin exerted its antiapoptotic effects by inhibition of free radicals, caspase‐3/‐7/‐9, hsp70, HMGB1, and p53, and the elevation of the antiapoptotic protein Bcl‐2. Rutin has potential therapeutic effectiveness against several toxicants, and its beneficial effects are more than likely mediated by its antioxidant, anti‐inflammatory, and/or antiapoptotic property.

## Graphical abstract

Rutin is a flavonoid present in many plant species. Because of its antioxidant, anti‐inflammatory, and anti‐apoptotic properties, rutin is of interest for its potential protective effects against toxic agents. Rutin has potential therapeutic effectiveness against several toxicants, and its beneficial effects are more than likely mediated by its antioxidant, anti‐inflammatory, and/or anti‐apoptotic property.

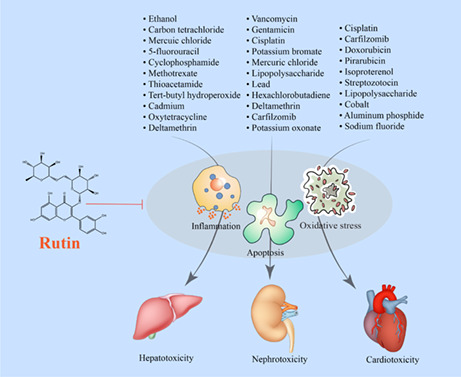

## 1 INTRODUCTION

**FIGURE 1:**
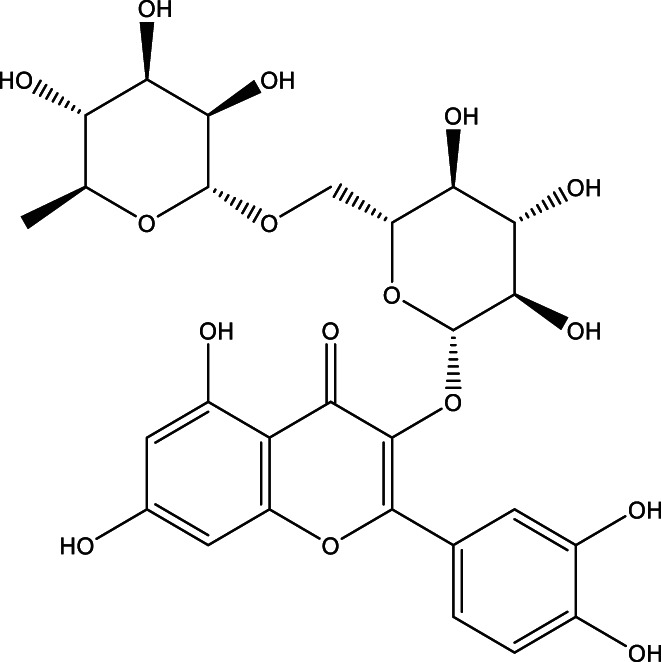
Chemical structure of the rutin

Over the past several decades, there has been an increased interest in natural products due, in no small part, to their radical scavenging, anti‐inflammatory, antidiabetic, and anticancer properties. The polyphenolic structure of flavonoids, a class of natural products, has been reported to be responsible for their therapeutic effects (Sharma et al., 2013; Yarmohammadi et al., 2021). Rutin (3′, 4′, 5, 7‐tetrahydroxyflavone‐3‐rutinoside, Figure 1) is one of the more interesting flavonoids found in plants like buckwheat, passionflower, tea, and apples (Frutos et al., 2019; Hosseinzadeh & Nassiri‐Asl, 2014; Rezvani et al., 2011). The concentration of rutin varies depending on the plant species, the plant part, and the geographical origin of the plant (Frutos et al., 2019).

Rutin exhibits protective effects on the heart (Li et al., 2020; Ma et al., 2017; Wang et al., 2017), the kidneys (Qu, Dai, Guo, et al., 2019; Qu, Dai, Lang, et al., 2019), and the liver (Lee et al., 2019; Zargar et al., 2017). The primary pharmacological effect and the underlying mechanism of rutin are enhancement of the antioxidant capacity via the nuclear factor–erythroid factor 2‐related factor 2/antioxidant response element (Nrf2/ARE); its anti‐inflammatory effect via suppression of nuclear factor‐kappa B (NF‐κB), tumor necrosis factor‐α (TNF‐α), cyclooxygenase 2 (COX‐2), and interleukin‐6 (IL‐6); and its antiapoptotic effect by inhibition of caspase‐3/−9 and B‐cell lymphoma 2 (Bcl‐2) enhancement (Janbaz et al., 2002; Nafees et al., 2015). Rutin has also been shown to modulate dynamin‐related protein 1 (DRP1), which is an essential protein involved in the regulation of mitochondrial fission (Choi et al., 2021). Rutin has metal‐chelating capabilities, thereby preventing metal ion‐induced peroxidation (Frutos et al., 2019). Rutin is hydrolyzed and converted into quercetin and rutinose by the gut microflora. According to one dietary supplement database, over 860 rutin‐containing products are available in the United States (Gullón et al., 2017). The potential protective effects of rutin against organ toxicity induced by both synthetic and natural compounds, with a focus on the liver, kidney, and heart as the major organs, will be reviewed.

## 2 METHODS

A comprehensive literature review was performed using the following keywords: “Rutin” and “Cardioprotective” OR “Hepatoprotective” OR “Nephroprotective” in the following databases: PubMed, Scopus, Medline, and Web of Science. All retrieved articles were reviewed to find relevant in vitro and in vivo studies published until February 2022. Only original articles were included. Duplicated, none relevant, and non‐English language articles were excluded. Following these search criteria, we found 65 articles in the online databases, among which 10 were excluded for the following reasons: no innovative or influential content and inconsistent or arbitrary conclusions; the remaining 55 articles were included in the current review.

### 2.1 The protective effects of rutin against hepatotoxicity

A major function of the liver is to detoxify chemicals. Drug‐ and chemical‐induced damage is a common cause of the liver disease (Gu & Manautou, 2012). Numerous substances have been shown to have hepatotoxic properties, including ethanol, carbon tetrachloride, and acetaminophen (Nathwani & Kaplowitz, 2006). The mechanisms by which chemicals cause liver damage include oxidative stress induction, the production of proinflammatory mediators such as TNF‐α, NF‐κB, COX, and nitric oxide (NO), and apoptosis. The hepatoprotective effect of rutin is attributed to its antioxidant, anti‐inflammatory, and antiapoptotic properties (Table 1).

**TABLE 1:** Summary of the rutin‐induced protective effects on the liver

Toxic agent	Dose/concentration, period, and route of exposure (toxic agent)	Dose/concentration, period, and route of exposure (rutin)	Experimental model	Results of rutin exposure	Reference
Ethanol	0%–2% for 1 day	0–10 μM for 1 day	HepG2 cells and zebrafish	Decreased DRP1 and hepatic steatosis, and provoked remodeling of mitochondrial dynamics	(Choi et al., 2021)
	20% for 60 days	25, 50, and 100 mg/kg for 30 days	Male albino rats	Decreased AST, ALT, ALP, and GGT Increased SOD, CAT, GPx, and GST	(Shenbagam & Nalini, 2011)
	4%–7% for 24 h	5, 10, 20 μM for 1 h	HepG2 cells	Decreased ALT, AST, ROS, MDA, ROS, and NO Increased GSH and upregulated Nrf2	(Lee et al., 2019)
Carbon tetrachloride	2 ml/kg, 10% *v*/*v*, for 5 days (intraperitoneal)	10, 50, and 150 mg/kg for 5 days (intraperitoneal)	BALB/c mice	Decreased AST, ALT, NF‐κB, TNF‐α, COX‐2, and TGF‐*β*1 Increased SOD, GSH, Nrf2, and HO‐1	(Domitrović et al., 2012)
	3 ml/kg for 4 weeks (intraperitoneal)	70 mg/kg for 4 weeks (oral)	Male Wistar rats	Decreased AST, ALT, IL‐6, Bcl‐xl, MEK5, FADD, EGF, STAT3, and JAK Increased Bcl‐2	(Hafez et al., 2015)
	3 ml/kg for 4 weeks (intraperitoneal)	70 mg/kg for 4 weeks (oral)	Male Wistar rats	Decreased LDL, MDA, H2O2, AST, ALT, LDL, cholesterol, TGs, and MCP‐1 Increased CAT, GST, GPX, HDL, PON‐1, PON‐3, ABCA‐1, and PPAR*δ*	(Hafez et al., 2014)
	3 ml/kg for 4 weeks (intraperitoneal)	50, 70 mg/kg for 4 weeks (oral)	Male Sprague–Dawley rat	Decreased ALT, AST, ALP, GGT, DNA fragmentation, and oxo8dG Increased SOD, CAT, GPx, GST, GR, GSH, p53, and CYP 2E1	(Khan et al., 2012a)
Mercuric chloride	1.23 mg/kg for 7 days	50 and 100 mg/kg for 1 week (oral)	Male Wistar rats	Decreased AST, ALP, ALT, and MDA Decreased EGFR, p53, p38 MAPK, Bax, procaspase‐3, cytochrome c, TNF‐α, IL‐1β, and NF‐κB Increased SOD, GPx, and GSH	(Caglayan, Kandemir, Darendelioğlu, et al., 2019)
5‐Fluorouracil	Single dose of 50 mg/kg	50 and 100 mg/kg, for 21 days (oral)	Male Sprague–Dawley rats	Decreased MDA, AST, ALT, LDH, ALP, and caspase‐3 Increased GPx, GSH, and Bcl‐2	(Gelen et al., 2017)
Thioacetamide	Single dose of 300 mg/kg (intraperitoneal)	10 mg/kg for 2 weeks (oral)	Male Wistar rats	Decreased AST, ALT, ALP, LDH, and DNA fragmentation	(Zargar et al., 2017)
Cadmium	150 mg/kg for 60 days (oral)	500 mg/kg for 60 days (oral)	Chicken	Decreased AST, ALT, MDA, ROS, NO, caspase‐8, iNOS, HSP27, HSP40, HSP60, HSP70, and HSP90 Increased CAT, SOD, GPx, JNK, ERK, P38, NF‐κB, TNF‐α, RIPK1, RIPK3, and MLKL	(Liu et al., 2022)
	Cd (50 mg/kg) + ethanol (5 mg/kg) for 15 days (oral)	25, 50, and 100 mg/kg for 2 weeks (oral)	Male Wistar rats	Decreased MDA, AST, ALT, ACP, GGT, cholesterol, and bilirubin Increased GSH, CAT, SOD, and GST	(Abarikwu et al., 2017)
Terutin‐butyl hydroperoxide	10 μM for 30 min and 2.5 mg/kg (intraperitoneal**)**	16.3 to 1.63 μM for 60 min 10, 50, and 100 mg/kg (oral)	Human erythrocyte and Swiss albino mice	Decreased ROS, MDA, TG, cholesterol, AST, ALT, ALP, bilirubin, and iNOS Increased GSH, CAT, GPx, GR, GST, SOD, and Nrf2	(Singh et al., 2019)
Methotrexate	Single dose of 20 mg/kg (intraperitoneal)	100 mg/kg (intraperitoneal)	Female Wistar rats	Decreased AST, ALT, and MDA Increased GSH and SOD	(Erdogan et al., 2015)
Cyclophosphamide	Single dose of 150 mg/kg (intraperitoneal)	50 and 100 mg/kg for 20 days (oral)	Male Wistar rats	Decreased AST, ALT, LDH TNF‐α, IL‐6, p38 MAPK, NF‐κB, iNOS, and COX‐2 Increased GSH, GPx, GR, CAT, XO, and LPO	(Nafees et al., 2015)
Deltamethrin	1.28 mg/kg (oral)	25 and 50 mg/kg for 30 days	Male Wistar rats	Decreased ALT, AST, ALP, TNF‐α, NF‐κB, IL‐1β, p38 MAPK, COX‐2, iNOS, beclin‐1, Bax, caspase‐3, PARP‐1, VEGF, and c‐fos Increased SOD, CAT, GPx, GSH, and Bcl‐2	(Küçükler et al., 2021)
Oxytetracycline	100 mg/kg for 2 weeks (oral)	1.5 g/kg for 2 weeks (oral)	Silver catfish	Decreased ALT, AST, MDA, HSP70, HMGB1, and caspase‐3 Increased SOD, CAT, GSH, GPx, GR, and G6PDH	(Londero et al., 2021)

#### 2.1.1 Ethanol

Chronic alcohol consumption is associated with liver disease, which can lead to a range of disorders such as steatosis, fibrosis, and carcinoma (Lee et al., 2019; Tang et al., 2013). Choi et al. (2021) demonstrated that rutin has a cytoprotective effect against hepatotoxicity induced by ethanol using HepG2 cells and zebrafish. The protective effect of rutin was associated with the inhibition of mitochondrial dynamics mediated by DRP1 in both zebrafish and HepG2 cells. DRP1 is a member of the dynamin GTPase superfamily localized at the mitochondrial outer membrane site, where it regulates the fission of mitochondria and other related cellular processes. Their results indicated that rutin reduced steatosis induced by ethanol in the liver of zebrafish larvae. Rutin has also been shown to decrease the levels of several hepatic enzymes including aspartate transaminase (AST), alkaline phosphatase (ALP), and gamma‐glutamyl transferase (GGT) in male rats. It reduced the ethanol‐induced hepatotoxicity by enhancing antioxidant enzymes including superoxide dismutase (SOD), catalase (CAT), glutathione peroxidase (GPx), and glutathione‐S‐transferase (GST), and non‐enzymatic antioxidants like glutathione (GSH). The hepatoprotective effect was confirmed by histological evaluation (Shenbagam & Nalini, 2011). The Nrf2/ARE pathway has been reported as an important signaling axis in preventing alcoholic liver damage. The protective effect of rutin against ethanol‐induced toxicity in HepG2 cells has been reported to involve the enhanced expression of the Nrf2/ARE pathway (Table 1) (Lee et al., 2019).

#### 2.1.2 Carbon tetrachloride

Carbon tetrachloride (CCl4) poisoning is a common experimental model for investigating hepatotoxicity (Weber et al., 2003). The hepatotoxic effect of CCl4 has been attributed to the excessive production of free radicals. It has been demonstrated that antioxidant‐rich substances reduced CCl4‐induced liver damage (Janbaz et al., 2002). Rutin administration in rats has been reported to ameliorate the oxidative stress induced by CCl4 via promoting CAT, SOD, GST, GSH‐Px, and GSH, and by suppressing lipid peroxidation. Rutin also mitigated DNA fragmentation and oxidative damage to the DNA caused by CCl4, as well as increased the expression of p53 and CYP2E1 (Khan et al., 2012a). A study in BALB/c mice suggested that rutin administration attenuated the inflammation caused by CCl4 via the downregulation of NF‐κB, TNF‐α, and COX‐2. Rutin also suppressed the expression of transforming growth factor‐β1 (TGF‐*β*1). Additionally, rutin treatment increased the expression of Nrf2 and heme oxygenase‐1 (HO‐1) in the injured liver. Histological examination confirmed that rutin prevented or at least reduced the hepatocellular damage caused by CCl4 (Table 1) (Hafez et al., 2014).

The inflammatory cytokine IL‐6 regulates multiple biological processes, including the induction of acute‐phase proteins in the liver (Hafez et al., 2015). Hafez et al. reported the hepatoprotective effect of rutin involved activation of the IL‐6/signal transducer and the activator of the transcription 3 (STAT3) pathway in CCl4‐induced hepatotoxicity in rats. These results indicated that rutin treatment reversed several biochemical markers of hepatotoxicity induced by CCl4 such as AST and ALT and downregulated the expression of IL‐6, B‐cell lymphoma–extra‐large (Bcl‐xl), mitogen‐activated protein kinase kinase 5 (MEK5), Fas‐associated via death domain (FADD), epidermal growth factor (EGF), STAT3, and Janus kinase (Domitrović et al., 2012). Moreover, rutin upregulated the expression of Bcl‐2 (Hafez et al., 2015). There is evidence that paraoxonases 1 and 3 (PON1 and PON3) reduce LDL oxidation. Low expression and activity of PON1 have been linked to chronic liver disease (Hafez et al., 2014). Hafez et al. have reported on the association between paraoxonase gene expression and oxidative stress in hepatotoxicity induced by CCl4. Their results indicated that rutin increased the expression of both PON1 and PON3. PPAR‐*δ* regulates several antioxidant genes, including SOD, CAT, and thioredoxin. Interestingly, rutin treatment increased the expression of ATP‐binding cassette transporter A1 (ABCA‐1), CAT, GST, and GPx, and lowered the expression of the monocyte chemoattractant protein‐1 (MCP‐1) gene (Table 1) (Hafez et al., 2014).

#### 2.1.3 Mercuric chloride

Mercury is a significant environmental pollutant that causes hepatotoxicity via oxidative stress (Palanisamy & Mohan Dass, 2013). Caglayan, Kandemir, Darendelioğlu, et al. (2019) reported that rutin treatment in rats reversed the hepatotoxicity induced by mercury chloride through the suppression of inflammatory parameters including NF‐κB, Bcl‐3, TNF‐α, IL‐1β, p53, and p38α mitogen‐activated protein kinase (MAPK) activity. Rutin also decreased mercury‐induced apoptosis by modulating the expression levels of proapoptotic Bcl‐2‐associated X protein (Bax), cytochrome c, and antiapoptotic Bcl‐2 and procaspase‐3. Moreover, the rate of Bax/Bcl‐2 induction was significantly decreased by rutin. Histopathological examination showed mild degeneration in the hepatocytes in the rutin‐treated rats with no necrosis, suggesting that rutin reduced the toxic effects of mercury in the liver (Table 1).

#### 2.1.4 5‐Fluorouracil

5‐Fluorouracil is an antimetabolic anticancer agent and influences the synthesis of DNA and RNA in both normal and tumor cells (Zhang et al., 2008). 5‐Fluorouracil exposure elevated liver enzymes such as AST, ALT, LDH, and ALP which were reversed by rutin. Moreover, rutin diminished malondialdehyde (MDA) levels and enhanced the GSH content and GPx activity. Rutin also modulated apoptotic markers by reducing caspase‐3 and increasing Bcl‐2. The arrangement of the hepatocytes in the liver was almost normal (Table 1) (Gelen et al., 2017).

#### 2.1.5 Cyclophosphamide

**FIGURE 2:**
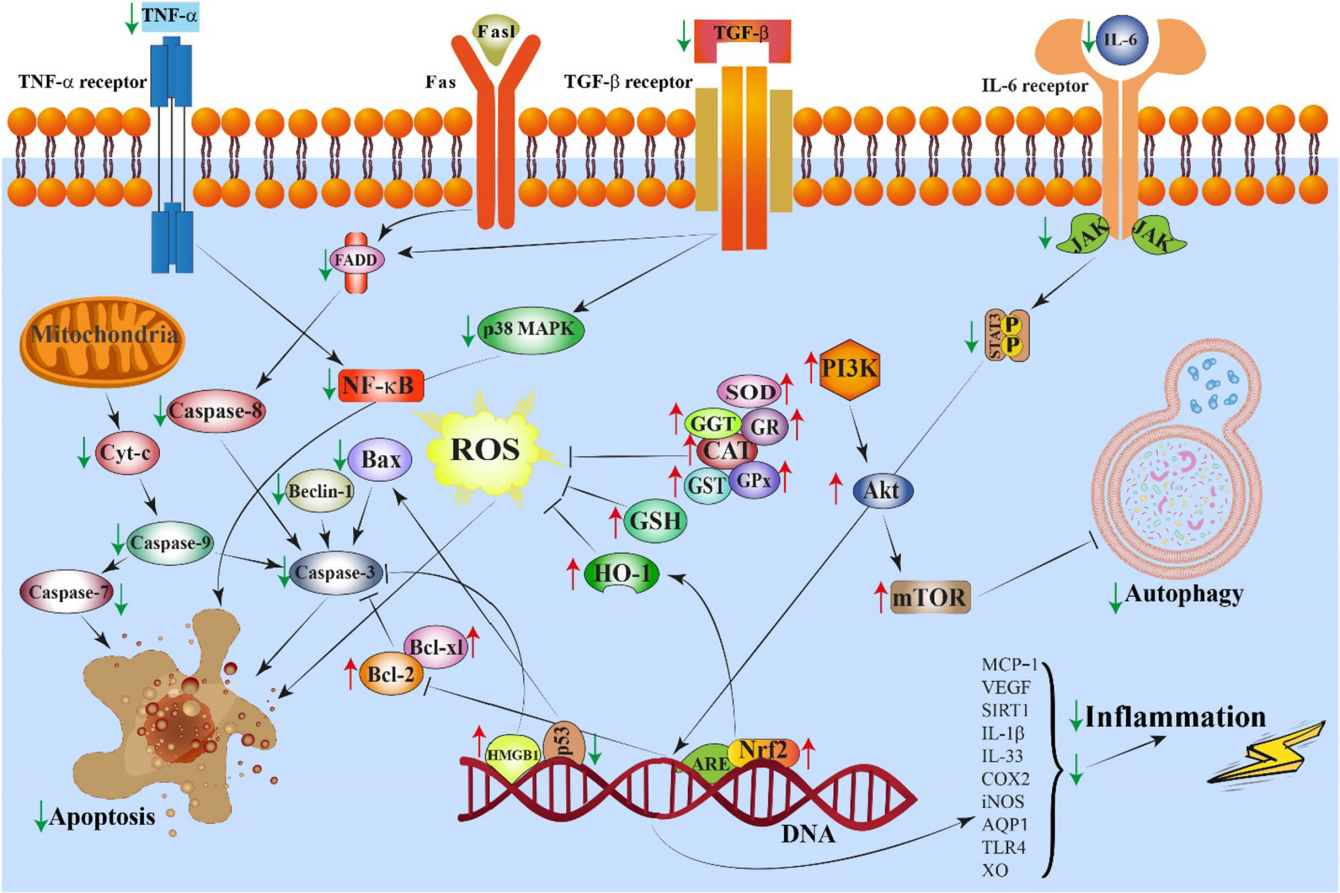
A schematic illustration of the rutin‐induced protective effects against toxic agents. ↑ and → present the promote/activate, ⊥ and ↓ present the inhibitory/suppressive effects

Cyclophosphamide is an anticancer medication used to treat a variety of human cancers and other diseases, including systemic lupus erythematosus, rheumatoid arthritis, and multiple sclerosis (Naicker et al., 2021). The use of cyclophosphamide, however, is restricted due to its toxicity including nephrotoxicity, hepatotoxicity, cardiotoxicity, and immunotoxicity Administration of rutin to male Sprague–Dawley rats ameliorated the cyclophosphamide‐induced hepatotoxicity by diminishing inflammatory markers such as TNF‐α, IL‐6, p38 MAPK, NF‐κB, inducible nitric oxide synthase (iNOS), and COX‐2 (Nafees et al., 2015). The histopathological findings confirmed these results (Nafees et al., 2015). The authors suggested that rutin might attenuate oxidative stress and inflammation induced by cyclophosphamide by suppressing the p38 MAPK/NF‐κB pathway (Table 1; Figure 2).

#### 2.1.6 Methotrexate

**FIGURE 3:**
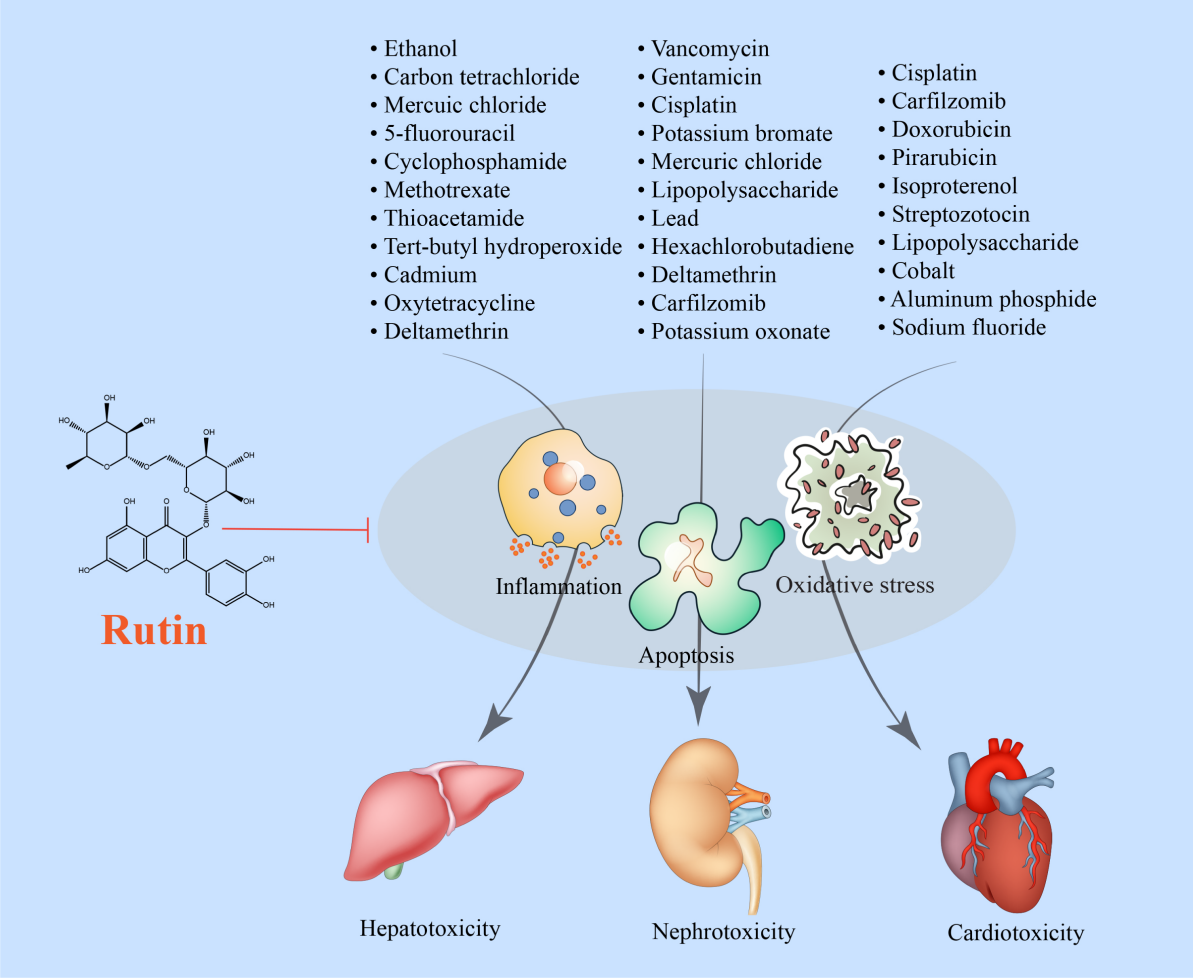
A schematic diagram of the rutin‐induced protective effects against toxic agents on the liver, kidneys, and heart. → and → present the promote/activate, ⊥ and ↓ present the inhibitory/suppressive effects

Methotrexate is a cytotoxic drug widely used in chemotherapeutic‐based cancer treatments. It has a noxious effect, however, on the liver, especially when given in high doses for long periods (Bath et al., 2014). A study by Erdogan et al. (2015) reported that methotrexate administration provoked liver toxicity reflected in elevated AST and ALT and decreased SOD and GSH‐Px. Rutin treatment reversed the effect induced by methotrexate. The hepatocyte vacuolization, sinusoidal dilation, and radial arrangement disruption induced by methotrexate in female Wistar rats were reversed by rutin (Table 1; Figure 3).

#### 2.1.7 Thioacetamide

Thioacetamide is an organosulfur chemical known to cause severe hepatotoxicity in animal models (Moustafa et al., 2014). In an in vivo study, rats exposed to thioacetamide had elevated AST, ALT, ALP, LDH, total bilirubin, and DNA fragmentation. Rutin administration before thioacetamide resulted in a significant reversal of liver damage. Histopathological examination of the liver showed no significant difference between the control and rutin‐treated animals (Table 1) (Zargar et al., 2017).

#### 2.1.8 Tert‐butyl hydroperoxide

Terutin‐butyl hydroperoxide (t‐BHP), an organic peroxide, is used to produce propylene oxide by the oxidation of propylene with tert‐butyl hydroperoxide. t‐BHP is also an environmental oxidative stress inducer that has been reported to generate ROS (Mir et al., 2021). Singh et al. (2019) evaluated the protective effects of rutin against t‐BHP in both in vivo and in vitro studies. The results indicated that rutin mitigated the toxicity induced by t‐BHP by modulating Nrf2 and iNOS. Rutin pretreatment modulated GSH, MDA, and CO levels. Rutin treatment also enhanced the activity of antioxidant enzymes such as CAT, SOD, GPx, and GST. Rutin, at the highest concentration, tested and protected erythrocytes by decreasing the level of ROS (Singh et al., 2019). Rutin exhibited its protective effect against t‐BHP‐induced toxicity via inhibition and activation of the iNOS and Nrf2 signaling pathways, respectively (Table 1) (Singh et al., 2019).

#### 2.1.9 Cadmium

Cadmium (Cd) is hepatotoxic to farm animals and humans (Ashrafizadeh et al., 2020). Liu et al. (2022) have reported that the protective effect of rutin against the hepatotoxicity induced by cadmium in chicken was mediated by inhibiting oxidative stress and the MAPK/NF‐κB pathway. Rutin treatment diminished the expression of JNKs, ERK, p38, NF‐κB, and TNF‐α and enhanced antioxidant enzymes like SOD, GPx, and CAT, while it decreased the oxidative stress biomarkers such as ROS, MDA, and NO. In addition, rutin treatment promoted the expression of HSP27, HSP40, HSP60, HSP70, and HSP90 (Liu et al., 2022). The protective effect of rutin has been associated with the necroptosis pathway, which is a pattern of regulated necrosis and different from apoptosis. Necroptosis has been reported to affect the liver during infections, inflammation, and hepatotoxicity. Rutin treatment regulated several signaling molecules related to this pathway, including the receptor‐interacting serine/threonine‐protein kinase 1/3 (RIPK1/3), mixed lineage kinase domain‐like protein (MLKL), and caspase‐8 (Table 1) (Liu et al., 2022).

Abarikwu et al. (2017) evaluated the protective effect of rutin against cadmium + ethanol‐induced hepatic toxicity in rats. Rutin treatment restored the activity of AST, ALT, ALP, LDH, and gamma‐glutamyl transferase (GGT). Rutin also restored the MDA, GSH, CAT, GSH‐Px, SOD, and GST levels. Animals treated with rutin and cadmium + ethanol had no visible liver lesions, while the animals treated with cadmium + ethanol showed severe hydropic degeneration of the hepatocytes (Table 1) (Abarikwu et al., 2017).

#### 2.1.10 Oxytetracycline

Oxytetracycline is a broad‐spectrum antibiotic used to treat various infections. Its usage can result in significant hepatotoxicity. Londero et al. (2021) showed that rutin administration reversed the hepatotoxicity induced by oxytetracycline in silver catfish by improving the oxidized glutathione (GSSG)‐to‐GSH ratio, G6PDH activity, and several other antioxidant enzymes such as SOD, GPx, and GR. These results indicated that rutin treatment promoted antiapoptotic effects via modulation of HSP70, HMGB1, and cleaved caspase‐3 expression (Table 1).

#### 2.1.11 Deltamethrin

Deltamethrin is a synthetic pyrethroid insecticide used worldwide to control mosquitoes, flies, and insects in general (Lu et al., 2019). Rutin exhibited a protective effect against deltamethrin‐induced liver toxicity. It decreased the levels of AST, ALT, and ALP. In addition, rutin treatment reduced the activation of inflammatory and protective‐apoptotic pathways by increasing Bcl‐2 and decreasing TNF‐α, NF‐κB, IL‐1β, p38 MAPK, COX‐2, iNOS, Beclin‐1, Bax, and caspase‐3 (Küçükler et al., 2021).

### 2.2 The protective effect of rutin against nephrotoxicity

A primary function of the kidney is homeostasis. In this regard, the kidneys control body fluid levels, electrolyte balance, acid–base balance, hormone secretion, blood pressure, and toxic metabolites excretion (Molaei et al., 2021). The high prevalence of acute renal failure in both hospitalized and nonhospitalized patients is often related to treatment with drugs (Al‐Harbi et al., 2019). Drug‐induced nephrotoxicity accounts for ~20% of hospital admissions for acute renal injury and up to 60% of in‐hospital acute kidney injuries (Jafari et al., 2013). Oxidative stress plays a crucial role in the mechanism of nephrotoxicity induced by various substances including drugs (Wu et al., 2018). Flavonoids like rutin are known to reverse oxidative stress. Rutin has antioxidant, anti‐inflammatory, and antiapoptotic *effects* against vancomycin, gentamicin, cisplatin, and cyclophosphamide (Table 2) (Qu, Dai, Lang, et al., 2019).

**TABLE 2:** Summary of the rutin‐induced protective effects on the kidney

Toxic agent	Dose/concentration, period, and route of exposure (toxic agent)	Dose/concentration, period, and route of exposure (rutin)	Experimental model	Results of rutin exposure	Reference
Vancomycin	200 mg/kg for 7 days (intraperitoneal)	150 mg/kg for 7 days (oral)	Male Wistar rats	Decreased NAG, BUN, Cr, MDA, NO, iNOS, caspase‐9, caspase‐3, IL‐1β, and TNF‐α Increased GSH, CAT, SOD, and Nrf2 mRNA	(Qu, Dai, Lang, et al., 2019)
	2 mM for 24 h	5, 10, and 20 μM	LLC‐PK1 cells	Decreased ROS, caspase‐3, caspase‐7, and caspase‐9 Increased SOD, CAT, and MMP	(Qu, Dai, Guo, et al., 2019)
Gentamicin	80 mg/kg for 8 days (intraperitoneal)	150 mg/kg for 6 days (oral)	Male Sprague–Dawley	Decreased MDA, CR, BUN, iNOS, cleaved caspase‐3, and LC3B Increased SOD, CAT, GPx, and GSH	(Kandemir et al., 2015)
Cisplatin	10 μg/ml for 24 h	12.5 and 25 μM for 1 h	Human mesangial cells	Decreased ROS, MDA, p53, cleaved caspase‐3, procaspase‐3, procaspase‐9 TNF‐α, NF‐κB, and IL‐1β Increased SOD	(Zhang, Wang, et al., 2019)
	Single dose of 7.5 mg/kg (intraperitoneal)	200 mg/kg/day for 10 days (oral)	Male Wistar rats	Decreased caspase‐3, cytochrome c, Cr, urea, MDA, TNF‐α, NF‐κB, and IL‐1β Increased GSH	(Radwan & Abdel Fattah, 2017)
	Single dose of 5 mg/kg (intraperitoneal)	30 mg/kg for 14 days (oral)	Male Wistar rats	Decreased BUN, Cr, MDA, IL‐1α, TNF α, p38, MKK4, MKK7, and JNK	(Alhoshani et al., 2017)
	7 mg/kg (intraperitoneal)	75 and 150 mg/kg for 21 days (oral)	Male Wistar rats	Decreased MDA, XO, H2O2, Cr, BUN, TNF‐α, NF‐κB, and caspase‐3 Increased GSH, GST, GR, and GPX	(Arjumand et al., 2011)
Potassium bromates	20 mg/kg for 4 weeks (oral)	50 mg/kg for 4 weeks (oral)	Male Sprague–Dawley rats	Decreased nitrite, Cr, BUN, MDA, AgNORs, and DNA injuries Increased CAT, SOD, GSH‐Px, GR, GST, total protein, albumin, and globulin	(Khan et al., 2012b)
Mercuric chloride	1.23 mg/kg for 7 days (intraperitoneal)	50 and 100 mg/kg for 7 days (oral)	Male Sprague–Dawley rats	Decreased MDA, urea, Cr, TNF‐α, Bcl‐3, IL‐1β, NF‐κB, IL‐33, MAPK 14, Bax, p53, caspase‐3, and 8‐OHdG Increased SOD, CAT, GPx, GSH, and AQP1	(Caglayan, Kandemir, Yildirim, et al., 2019)
Lead acetate	30 mg/kg for 7 days (oral)	50 mg/day for 7 days (oral)	Male Sprague–Dawley rats	Decreased BUN, Cr, and uric acid Increased GSH, CAT, GPx, and SOD	(Ansar et al., 2016)
Deltamethrin	1.28 mg/kg for 30 days (oral)	25 and 50 mg/kg for 30 days (oral)	Male Sprague–Dawley rats	Decreased ALT, AST, ALP, urea, Cr, TNF‐α, NF‐κB, IL‐1β, p38 MAPK, COX‐2, iNOS, beclin‐1, Bax, and caspase‐3 Increased SOD, CAT, GPx, and GSH	(Küçükler et al., 2021)
Carfilzomib	4 mg/kg, for 3 weeks (intraperitoneal)	10 mg/kg for 3 weeks (oral)	Male Wistar rats	Decreased Cr, BUN, MDA, NOS‐2, NF‐κB, IkBa, IL‐17, caspase‐3, and p65 Increased RBCs, WBCs, platelets, Hb, HCT, GSH, CAT, and GR	(Al‐Harbi et al., 2019)
Hexachlorobutadiene	Single dose of 100 mg/kg (intraperitoneal)	100, 500, and 1000 mg/kg (intraperitoneal)	Female Wistar rats	Decreased MDA, urea, Cr, and urine glucose and protein Increased total thiol	(Sadeghnia et al., 2013)
Lipopolysaccharide	Single dose 10 mg/kg, (intraperitoneal)	Single doses of 50 and 200 mg/kg (oral)	Female C57BL/6 mice	Decreased Cr, BUN, MDA, COX‐2, TLR4, SIRT1, TNF‐α, caspase‐3, and IL‐6 Increased SOD and GSH	(Khajevand‐Khazaei et al., 2018)
Potassium oxonate	250 mg/kg for 7 days (oral)	25, 50, and 100 mg/kg for 7 days (oral)	Male Kunming mice	Decreased Cr, BUN, uric acid, mGLUT9, and mURAT1 Upregulated mOAT1, mOCT1‐2, and urinary uromodulin	(Chen et al., 2013)

#### 2.2.1 Vancomycin

Vancomycin is an antibiotic used to treat gram‐positive bacterial infections. Vancomycin, however, can cause nephrotoxicity (Brunetti et al., 2020). In a study conducted by Qu et al., male Wistar rats were exposed to vancomycin and rutin. Co‐administration of the two chemicals induced a significant suppression in blood urea nitrogen (BUN), creatinine (Savi et al., 2017), and *N*‐acetyl‐beta‐d‐glucosaminidase (NAG), all of which were elevated in the vancomycin‐treated animals. Co‐administration also restored several renal functional markers (Qu, Dai, Lang, et al., 2019). The histopathology supported the conclusion that rutin improved vancomycin‐induced tissue damage. Vancomycin‐induced renal oxidative stress was reflected by modeling oxidative and nitrosative stress markers such as MDA, GSH, CAT, SOD, NO, and iNOS (Qu, Dai, Lang, et al., 2019). Rutin reduced vancomycin‐induced activation of caspase‐9, caspase‐3, IL‐1β, and TNF‐α and upregulated the Nrf2 mRNA level and the respective downstream target gene, *HO‐1*. The nephroprotective effect of rutin was associated with the upregulation of the Nrf2/HO‐1 pathway (Table 2) (Qu, Dai, Lang, et al., 2019).

The effect of rutin on vancomycin‐induced oxidative stress, mitochondrial dysfunction, and apoptosis was investigated in LLCPK1 renal tubular epithelial cells. The results indicated that rutin pretreatment ameliorated vancomycin‐induced toxicity by suppressing oxidative stress. The mitochondrial membrane potential alteration caused by vancomycin was reduced in a dose‐dependent manner by rutin. Rutin also promoted antiapoptotic effects by downregulating caspase‐3/7 and ‐9 activities in vancomycin‐treated LLC‐PK1 cells (Table 2) (Qu, Dai, Guo, et al., 2019).

#### 2.2.2 Gentamicin

Gentamicin is an antibiotic that is useful in treating severe sepsis. However, its usage is restricted due to the development of ototoxicity and nephrotoxicity (Morin et al., 1980). Gentamicin‐induced nephrotoxicity is related to oxidative stress and inflammatory cascades (Sahu et al., 2014). In an in vivo study, rutin decreased gentamicin‐induced nephrotoxicity by diminishing MDA levels while enhancing SOD, CAT, and GPx activities, as well as GSH levels. The suppression of iNOS, cleaved caspase‐3, and light chain 3B (LC3B by rutin was confirmed by immunohistochemical staining). Rutin also exhibited antioxidant, anti‐inflammatory, antiapoptotic, and antiautophagy properties (Table 2) (Kandemir et al., 2015).

#### 2.2.3 Cisplatin

Cisplatin is used to treat head, ovarian, neck, colon, and testicular cancers. However, the use of cisplatin is limited due to its side effects, such as nephrotoxicity, ototoxicity, neurotoxicity, and cardiomyopathy (Arjumand et al., 2011). Zhang et al. reported that rutin reversed the cisplatin‐induced upregulation of p53, cleaved‐caspase‐3, and suppressed procaspase‐3/9 levels in human mesangial cells. Pretreatment with rutin attenuated both oxidative stress and cell death by decreasing ROS and MDA. The protective effect of rutin has been reported to be mediated through the p53 signaling pathway (50). Co‐exposure of rutin and low‐dose gamma irradiation 2 day before cisplatin administration reversed the overall toxicity as measured by a decrease in the oxidative biomarkers, MDA and GST, and the elevated serum nephrotoxic markers Cr and urea in rats.

The anti‐inflammatory properties of rutin have been associated with the suppression of TNF‐α, NF‐κB, and IL‐1β. Moreover, its antiapoptotic properties were induced via the reduction in cytochrome c and caspase‐3. These results were confirmed by histological assessments (Alhoshani et al., 2017; Radwan & Abdel Fattah, 2017). Another in vivo study revealed that administration of rutin improved cisplatin‐induced nephrotoxicity through the activation of the p38 MAPK pathway via modulation of mitogen‐activated protein kinases 4/7 (MKK4/7), JNK, p38, TNF‐α, IL‐1α, and TNF receptor‐associated factor 2 (TRAF2). These results suggested that the overexpression of TRAF2 may be attributed to the nephrotoxicity and the apoptosis induced by cisplatin. Rutin exerted its protective effect by regulating p38 MAPK and the apoptotic pathway (Table 2) (Alhoshani et al., 2017).

In a similar study, Arjumand et al. (2011) assessed the protective effects of rutin against the nephrotoxicity induced by cisplatin in rats. Rutin improved cisplatin‐induced lipid peroxidation, xanthine oxidase (XO) activity, GSH depletion, and decreased antioxidant enzyme activity (CAT, GR, GGT, and GPx). In addition, rutin diminished the expression of caspase‐3, TNF‐α, and NF‐κβ proteins. The rutin‐treated group had lowered BUN and Cr with fewer histological findings (Table 2).

#### 2.2.4 Potassium bromates

Potassium bromate is a food additive and a by‐product of water disinfection. In both humans and experimental animals, potassium bromated has been shown to induce multiorgan toxicities (Shanmugavel et al., 2020). The protective effect of rutin against potassium bromate toxicity is mediated through the antioxidant properties of rutin (Khan et al., 2012b). Potassium bromate has been shown to significantly decrease the activity of antioxidant enzymes like CAT, SOD, GSH‐Px, GR, and GST. Cotreatment of rutin with potassium bromate restored the activity of these antioxidant enzymes, and serum nitrite, Cr, BUN, total protein, and albumin returned to normal levels. Lipid peroxidation, argyrophilic nucleolar organizer region (AgNOR) count, and DNA damage were improved by the administration of rutin. Histological examination showed tubular degeneration, tubular dilation, and glomerular damage in animals treated with potassium bromate. These changes were reversed by rutin treatment (Table 2) (Khan et al., 2012b).

#### 2.2.5 Mercuric chloride

Mercury is a hazardous environmental and industrial contaminant that occurs in a range of chemical forms, including elemental, organic, and inorganic mercury (Bernhoft, 2012). Enhancement of MDA levels, suppression of antioxidant enzyme activities such as CAT, SOD, GPx, and GSH, and production of inflammatory factors like TNF‐α, Bcl‐3, IL‐1β, NF‐κB, and IL‐33 have all been reported to be involved in mercury‐induced nephrotoxicity in rats. Rutin has been shown to completely or partially reverse these changes (Figure 2). Rutin has also been reported to enhance the activities of MAPK 14 and myeloperoxidase (Caglayan, Kandemir, Yildirim, et al., 2019). Mercury‐induced apoptosis has been reported to be reversed by rutin supplementation through the mitigation of oxidative stress, apoptosis, and inflammation (Table 2) (Caglayan, Kandemir, Yildirim, et al., 2019).

Aquaporins (AQPs) are membrane protein channels involved in water transfer and, in some cases, the migration of small ions across cell membranes (Caglayan, Kandemir, Yildirim, et al., 2019). Rutin treatment markedly alleviated the decrease in AQPs levels (Caglayan, Kandemir, Yildirim, et al., 2019). Rutin has been shown to reduce 8‐OHdG, an important biomarker of oxidative DNA damage (Caglayan, Kandemir, Yildirim, et al., 2019). The mercury‐treated rats had cystic dilations, hydropic degeneration, and coagulation necrosis in their renal tubules which were partially reversed by rutin (Table 2) (Caglayan, Kandemir, Yildirim, et al., 2019).

#### 2.2.6 Lipopolysaccharide

Lipopolysaccharide (LPS) is the major component of the outer membrane of gram‐negative bacteria that causes strong inflammatory and immunological responses in animals and is thought to be implicated in the pathophysiology of sepsis‐induced acute kidney injury (Nozaki et al., 2020). It has been shown that rutin administration restored kidney injury induced by LPS in C57BL/6 mice as assessed by lower serum levels of Cr and BUN. The nephroprotective effect of rutin against LPS was reported to be mediated through the inhibition of toll‐like receptor 4 (TLR4), COX‐2, sirtuin 1 (Sirt1), TNF‐α, IL‐6, and caspase‐3 (Table 2) (Khajevand‐Khazaei et al., 2018).

#### 2.2.7 Lead

Lead is a heavy metal contaminant with multiple industrial uses. Exposure to lead can cause a variety of adverse effects including nephrotoxicity resulting in substantial changes in the structure and function of the kidneys (Shafiekhani et al., 2019). It has been reported that pretreatment of male Sprague–Dawley rats with rutin elicited a protective effect against lead‐induced toxicity by elevating antioxidant parameters such as GSH, CAT, GPx, and SOD (Ansar et al., 2016). Additionally, following rutin treatment in lead acetate‐treated rats, there was a decline in BUN, Cr, and uric acid, which was increased following lead acetate exposure only in rats (Table 2) (Ansar et al., 2016).

#### 2.2.8 Hexachlorobutadiene

Hexachlorobutadiene is a halogenated aliphatic chemical used in the industry for elastomers, rubber, heat‐transfer liquids, transformers, hydraulic fluids, and insecticide, herbicide, and fungicide formulations. Rutin administration 1 h before hexachlorobutadiene injection reversed the nephrotoxicity as reflected in a decrease in lipid peroxidation, serum Cr, urea, and both urine glucose and protein in female Wistar rats (Table 2) (Sadeghnia et al., 2013).

#### 2.2.9 Deltamethrin

Deltamethrin is a synthetic pyrethroid insecticide (Lu et al., 2019). The administration of deltamethrin has been shown to induce hepatotoxicity and nephrotoxicity in rats (Küçükler et al., 2021). Pretreatment with rutin reversed the deltamethrin‐induced toxicity by decreasing TNF‐α, IL‐1β, p38 MAPK, COX‐2, and iNOS in the liver and kidneys, again reflecting the anti‐inflammation property of rutin. Rutin also decreased the mRNA expression levels of PARP‐1, vascular endothelial growth factor (VEGF), and the immunohistochemical expression of c‐fos (Table 2) (Küçükler et al., 2021).

#### 2.2.10 Carfilzomib

Carfilzomib (CFZ) is a U.S. FDA‐approved proteasome enzyme inhibitor used to treat multiple myeloma. CFZ, however, is nephrotoxic with an unknown mechanism(s) (Al‐Harbi et al., 2019). CFZ exposure increases white blood cells, hemoglobin, and hematocrit and decreases antioxidant enzymes (Al‐Harbi et al., 2019). Al‐Harbi et al. (2019) reported that the protective effect of rutin on CFZ‐induced renal damage in rats was mediated via oxidative stress and inflammation inhibition. These effects were reversed by rutin administration (Al‐Harbi et al., 2019). In addition, the nephrotoxicity induced by CFZ increased caspase‐3 enzyme activity, implying the induction of apoptosis. Rutin showed a significant antiapoptotic effect via inhibition of caspase‐3 (Table 2) (Al‐Harbi et al., 2019).

#### 2.2.11 Potassium oxonate

In nonclinical studies, potassium oxonate is utilized to induce hyperuricemic animal models (Chen et al., 2013). Chen et al. (2013) investigated the putative potential of rutin to improve renal function via the modulation of the renal organic ion transporters in a hyperuricemic animal model. Their results showed that rutin administration reversed the oxonate‐induced hyperuricemia. Moreover, rutin downregulated renal mRNA and the levels of the glucose transporter 9 (mGLUT9) and the urate transporter 1 (mURAT1) but upregulated the renal organic anion transporter 1 (mOAT1) in hyperuricemic mice. Rutin treatment also upregulated the renal organic cation/carnitine transporters (mOCT1 and mOCTN2) and decreased the serum and kidney uromodulin levels that correlated with improved renal function. In the hyperuricemic mice model, renal mURAT1, mGLUT9, mOAT1, mOCT1‐2, and uromodulin contributed to rutin‐mediated uricosuric and protective effect on the kidneys (Table 2).

### 2.3 The protective effect of rutin against cardiotoxicity

Cardiotoxicity is the alteration, injury, and/or dysfunction of the heart that may develop following drug, heavy metal, pesticide, or toxicant exposure. Various mechanisms have been suggested for chemicals‐induced cardiotoxicity, including apoptosis induction, cytokines release, ROS generation, and suppression of the antioxidant system (El‐Nahhal & El‐Nahhal, 2021; Zhang, Zhu, et al., 2019). Due to its anti‐inflammatory, antiapoptosis, and antioxidant properties, rutin may have the potential to alleviate some of the cardiotoxicity caused by such agents (Table 3).

**TABLE 3:** Summary of rutin protective effects on the heart

Toxic agent	Dose/concentration, period, and route of exposure (toxic agent)	Dose/concentration, period, and route of exposure (rutin)	Experimental model	Results of rutin exposure	Reference
Cisplatin	5 mg/kg, every 2 days for 8 days (intraperitoneal)	100 mg/kg for 8 days (oral)	Male Wistar rats	Decreased MDA, IL‐1β, TNF‐α, troponin I, and CK Increased GSH	(Topal et al., 2018)
Carfilzomib	4 mg/kg for 16 days (intraperitoneal)	40 mg/kg, for 16 days (oral)	Male Wistar rats	Decreased NF‐ĸB, MDA, β‐MHC, and BNP mRNA expression. Increased α‐MHC and GSH	(Waxman et al., 2018)
Doxorubicin	3 mg/kg for 2 weeks (intraperitoneal)	100 mg/kg for 11 weeks (oral)	Male C57BL/6J mice	Decreased cardiac fibrosis, LC3 II, ATG5, P62 Bax, and caspase‐3 Increased Bcl‐2	(Ma et al., 2017)
	Single dose of 15 mg/kg (intraperitoneal)	100 μmol/kg for 5 days (intraperitoneal)	Male CDF1 mice	Decreased LPO and CPK activities Increased GSH‐px	(Sadzuka et al., 1997)
Pirarubicin	Single dose of 18 mg/kg (intravenous)	50 mg/kg for 7 days (oral)	Male Wistar rats	Decreased MDA, BNP, CK‐MB, cTnT, LDH, Bax, JNK, and caspase‐3, Increased SOD and Bcl‐2.	(Wang et al., 2018)
	5 μM for 24 h	100 μM for 24 h	HL‐1 cell line	Decreased ROS and miR‐125b‐1‐3p Increased JunD signaling	(Li et al., 2020)
	5 μM for 24 h	50 μM for 24 h	H9c2 cell line	Decreased NF‐κB, Bax, caspase‐3, and caspase‐9 Increased PI3K/Akt/mTOR activity, VEGF, and FGF1	(Fei et al., 2019)
	5 μM for 24 h	50 μM for 24 h	H9c2 cell line	Decreased TGF‐*β*1, p38 MAPK, caspase‐9, caspase‐7, and caspase‐3 Increased cell viability	(Wang et al., 2017)
Isoproterenol	Two doses of 100 mg/kg (intraperitoneal)	80 mg/kg for 42 days (oral)	Male Wistar rats	Increased SOD, CAT, GPx, GSH, ICDH, SDH, MDH, a‐KGDH, and NADH function.	(Punithavathi et al., 2010)
	Two doses of 150 mg/kg (intraperitoneal)	80 mg/kg for 42 days (oral)	Male Wistar rats	Decreased total cholesterol, TG, FFA, LDH, AST, ALT, and Ca^2+^ATPase activity Increased Na^+^/K^+^ and Mg^2+^ ATPases	(Karthick & Stanely Mainzen Prince, 2006; Stanely Mainzen Prince & Karthick, 2007)
Streptozotocin	Single dose of 45 mg/kg (intraperitoneal)	50 mg/kg for 42 weeks (oral)	Female Wistar rats	Decreased blood glucose HbA1c, TNF‐α, CRP, and BNP expression.	(Saklani et al., 2016)
	Single dose of 45 mg/kg (intraperitoneal)	10 mg/kg (intraperitoneal)	Male and female Wistar rats	Decreased MDA Increased SOD and CAT	(Annapurna et al., 2009)
Lipopolysaccharide	Single dose of 8 mg/kg (intraperitoneal)	100 mg/kg for 8 days (oral)	Male BALB/c mice	Decreased CK, MDA, LDH, MMP‐9/2, TNF‐α, and IL‐6 Increased SOD and CAT	(Xianchu et al., 2018)
Cobalt	60 μg for 24 h	120 μg 24 h	H9c2 cell line	Decreased MDA, LDH activity, HIF‐1α, Bax, and caspase‐3 Increased cell viability and Bcl‐2	(Sundaram et al., 2018)
Aluminum phosphide	Single dose of 2 mg/kg (oral)	100 mg/kg	Male Wistar rats	Decreased MDA, caspase‐3, TNF‐α, and IL‐6 Increased SOD and GSH	(El Wafa & El Noury, 2020)
Sodium fluoride	600 ppm for 21 days (oral)	50 mg/kg for 21 days (oral)	Male and female Wistar rats	Decreased LPO, TC, LDL, and TG Increased HDL, hemoglobin, RBC, and WBC	(Umarani et al., 2015)

#### 2.3.1 Cisplatin

Cisplatin is an anticancer drug used to treat a variety of cancers. However, its application has been reported to induce several toxicities including nephrotoxicity and cardiotoxicity (Karimi et al., 2005). Rutin suppressed MDA, IL‐1β, TNF‐α, troponin I, creatine kinase (CK), and CK‐MB levels and restored GSH content in the serum and cardiac tissue of cisplatin‐treated rats (Table 3) (Topal et al., 2018).

#### 2.3.2 Carfilzomib

Carfilzomib is a second‐generation proteasome inhibitor with minimal off‐target toxicities, including hepatotoxicity, cardiotoxicity, and nephrotoxicity (Waxman et al., 2018). Imam et al. demonstrated that rutin prevented the cardiac damages induced by carfilzomib through the suppression of NF‐ĸB, the slower MHC motor protein (β‐MHC), and BNP mRNA expression while increasing the faster MHC motor protein (α‐MHC) mRNA expression, key biomarkers of cardiotoxicity (Barry et al., 2008). Rutin also abolished MDA but enhanced the GSH content (Table 3) (Imam et al., 2017).

#### 2.3.3 Doxorubicin

Doxorubicin is an antineoplastic drug used to treat a range of malignancies such as carcinomas, sarcomas, and hematological cancers. However, doxorubicin can induce cardiotoxicity (Koleini & Kardami, 2017). Rutin administration to mice exposed to doxorubicin improved the left ventricular ejection fraction (EF) and mitigated or at least reduced cardiac fibrosis, LC3 II, ATG5, and P62 expressions, and autophagic markers (Roohbakhsh et al., 2016). Rutin also reduced the apoptotic markers, Bax and caspase‐3, and increased antiapoptotic Bcl‐2 (Ma et al., 2017). Sadzuka et al. (1997) demonstrated that rutin administration to mice prevented doxorubicin cardiotoxicity through enhancement of GSH‐Px activity, and suppression of lipid peroxidation and creatine kinase (CPK) in heart tissue (Table 3).

#### 2.3.4 Pirarubicin

Pirarubicin is a member of a group of cell cycle nonspecific anthracycline anticancer drugs. Cardiac toxicity, alopecia, and digestive system disturbance have been reported following treatment with pirarubicin (Wang et al., 2020). Rutin decreased the pirarubicin‐induced electrocardiogram abnormalities and cardiac dysfunction in rats. The serum levels of MDA, B‐type natriuretic peptide (BNP), CK‐MB, cardiac troponin T (cTnT), LDH, and SOD were attenuated following rutin exposure in pirarubicin‐treated rats. Rutin also increased the Bcl‐2/Bax ratio and suppressed the JNK and caspase‐3 protein content in cardiac tissue (Wang et al., 2018). JNK is a key protein in the MAPK pathway that plays an important role in the cell cycle, apoptosis, and cell stress (Lou et al., 2005). Rutin treatment of HL‐1 cells exposed to pirarubicin reduced oxidative stress. In addition, rutin activated the JunD signaling pathway by downregulating miR‐125b‐1‐3p expression that plays an essential role in cancer progression and immunosuppression (Li et al., 2020). JunD is a transcription factor that belongs to the activating protein‐1 (AP‐1) family and serves as an activator or inhibitor of the expression of multiple genes (Good et al., 2019).

In H9c2 cells treated with pirarubicin, rutin activated the PI3K/Akt/mTOR signaling pathway and attenuated NF‐κB expression, thereby activating several antioxidant enzymes and the angiogenesis‐promoting factors VEGF and FGF1 (Fei et al., 2019). Rutin inhibited apoptosis through the upregulation of Bcl‐2 and the downregulation of Bax, caspase‐3, and caspase‐9 in H9c2 cells (Fei et al., 2019). Treatment of H9c2 cardiomyoblasts with rutin prevented the pirarubicin‐induced reduction in cell viability and at the same time upregulated TGF‐*β*1, p38 MAPK, cleaved caspase‐9, caspase‐7, and caspase‐3 (Figure 2). These results suggested that rutin promoted its cardioprotective effects through the inhibition of ROS generation and the reduction of cell apoptosis by the TGF‐*β*1/p38 MAPK signaling pathway (Table 3) (Wang et al., 2017).

#### 2.3.5 Isoproterenol

Isoproterenol is a potent nonselective beta‐adrenergic agonist that is used to induce myocardial infarction in animal models (Sood et al., 2005). Treatment of rats with rutin prevented the isoproterenol‐induced cardiotoxicity by increasing SOD, CAT, GPx, and GSH and reducing LDH activity, thereby regulating isocitrate dehydrogenase (ICDH), mitochondrial succinate dehydrogenase (SDH), malate dehydrogenase (MDH), α‐ketoglutarate dehydrogenase (α‐KGDH), nicotinamide adenine dinucleotide dehydrogenase, and cytochrome‐c‐oxidase (Punithavathi et al., 2010). Isoproterenol‐treated rats have elevated heart weight, total serum cholesterol, triglycerides (TGs), free fatty acids, and reduced phospholipid concentrations that were reversed following rutin administration (Punithavathi et al., 2010). Rutin also enhanced the activity of sodium potassium‐dependent adenosine triphosphatase (Na^+^/K^+^ ATPase) and magnesium‐dependent adenosine triphosphatase (Mg^2+^ ATPase) while at the same time, decreasing the activity of calcium‐dependent adenosine triphosphatase (Ca^2+^ ATPase), CPK, LDH, AST, and ALT in cardiac tissue (Karthick & Stanely Mainzen Prince, 2006; Stanely Mainzen Prince & Karthick, 2007). Rutin was reported to restore isoproterenol‐induced elevated ROS, suppress antioxidant enzymes, and increase cTnT in the blood in another investigation (Table 3) (Filipský et al., 2017).

#### 2.3.6 Streptozotocin

Streptozotocin (STZ) can be used to induce experimental diabetes and cardiotoxicity (Umbarawan et al., 2020). STZ exposure produces cardiomyopathy through suppression of the antioxidant capacity and the enhancement of inflammatory cytokines in rats (Saklani et al., 2016). These changes were ameliorated by rutin treatment (Saklani et al., 2016). Treatment with rutin also ameliorated ischemia/reperfusion‐induced myocardial infarction, reduced MDA, increased SOD and CAT, and regulated heart rate in STZ‐treated rats (Table 3) (Annapurna et al., 2009).

#### 2.3.7 Lipopolysaccharide

Lipopolysaccharide has been reported to cause organ damage including the heart and brain by induction of an inflammatory response (Li et al., 2019). Cardiotoxicity resulting from exposure to LPS has been hypothesized to involve several mechanisms, including enhancement of matrix metalloproteinases 2 and 9 (MMP‐2 and ‐9), proinflammatory cytokines (TNF‐α and IL‐6), and suppression of antioxidant enzymes (SOD and CAT). These changes were reversed by rutin pretreatment in male mice (Table 3) (Xianchu et al., 2018).

#### 2.3.8 Cobalt

Cobalt is an essential trace element with both industrial and biological applications. However, excessive concentrations of cobalt are known to elicit cardio‐renal dysfunctions (Linna et al., 2004). Rutin prevented cobalt‐induced toxicity in H9c2 cells by enhancing cell viability, reducing MDA concentration, LDH activity, and downregulating hypoxia‐inducible factor 1‐alpha (HIF‐1α), Bax, and caspase‐3, while upregulating Bcl‐2 expression. These effects of rutin were mediated by the modulation of Akt and p38 and GSH and SOD enhancement (Table 3) (Sundaram et al., 2018).

#### 2.3.9 Aluminum phosphide

Aluminum phosphide, a pesticidal fumigant, has been shown to induce cardiotoxicity by generating oxidative stress and mitochondrial damage (Hosseini et al., 2020). Rats exposed to aluminum phosphide had suppressed heart rate and ST elevation, and increased GSH, TNF‐α and IL‐6, MDA and caspase‐3 levels, and SOD activity. Rutin ameliorated these changes (Table 3) (El Wafa & El Noury, 2020).

#### 2.3.10 Sodium fluoride

Barbier et al. (2010) reported that sodium fluoride exposure resulted in lower cognitive function ability, intelligence quotient, mental disorders, and cardiovascular and hepatic toxicities. Umarani et al. (2015) demonstrated that sodium fluoride treatment elevated lipid peroxidation, total cholesterol, TGs, white blood cells, and erythrocyte sedimentation and decreased high‐density lipoprotein (HDL), hemoglobin, red blood cells, mean corpuscular volume, and mean corpuscular hemoglobin in rats. These alterations were reversed by rutin (Table 3).

## 3 DISCUSSION AND CONCLUSION

Rutin has various pharmacological effects. In the present article, we have reviewed studies dealing with the protective effects of rutin on the heart, liver, and kidneys. Previous studies have shown that rutin can induce significant protective effects against synthetic chemicals and toxins with diverse mechanisms of toxicities. The beneficial effects of rutin are mainly associated with its antioxidant, anti‐inflammatory, and antiapoptotic effects. The antioxidant effect of rutin has been reported to involve enhancing the activity of enzymes such as SOD, GST, GGT, CAT, and GPx GR, and inducing the Nrf2/HO‐1 pathway. The anti‐inflammatory effects of rutin are mediated by the inhibition of well‐known proinflammatory mediators and molecules such as IL‐1β, IL‐6, TGF‐β1, COX‐2, iNOS, TLR4, and XO. Inflammation and oxidative stress are processes that predispose tissues to malfunction and may end with the destruction of the target tissue. So, reduction in inflammation and oxidative stress reduction are key interventions in alleviation of such toxic effects.

In addition, rutin has been reported to exert its antiapoptotic effects by inhibiting apoptotic molecules such as caspase‐3/‐7/‐9, and the elevation of antiapoptotic proteins including HMGB1 and Bcl‐2. The beneficial effects of rutin on the heart, liver, and kidneys have been confirmed by histopathological examinations in a variety of studies. Rutin elicited significant cardioprotective, nephroprotective, and hepatoprotective effects that were mainly mediated by its antioxidant and anti‐inflammatory properties. Considering these findings, further evaluation of this natural compound's ability to overcome the toxic effects of various toxicants is recommended.

This review summarized the findings of several important studies focusing on the protective effects of rutin against chemical and natural toxins. Based on the results, rutin could be a promising component in the prevention and treatment of several toxicities, including hepatotoxicity, nephrotoxicity, and cardiotoxicity, and protect organs against many agents that produce free radicals. Although the biological effects of rutin on ROS‐induced skin aging have been reported in a human study, further research on the protective effects of rutin and its mechanisms in human are needed to confirm animal studies. Furthermore, some of the studies discussed in this review article did not follow a recent document outlining the best practices for pharmacological research on herbal bioactive preparations.

## CONFLICT OF INTEREST

The authors declare that they have no conflict of interest.

## Data Availability

Data available on request from the authors.
